# Sympathetic Overactivation From Supine to Upright Is Associated With Orthostatic Hypertension in Children and Adolescents

**DOI:** 10.3389/fped.2020.00054

**Published:** 2020-02-21

**Authors:** Yang Hu, Yuanyuan Wang, Bing He, Yaru Wang, Zhenhui Han, Chunyan Tao, Hongxia Li, Yi Jiang, Chaoshu Tang, Junbao Du

**Affiliations:** ^1^Department of Pediatrics, Peking University First Hospital, Beijing, China; ^2^Research Unit of Clinical Diagnosis and Treatment of Pediatric Syncope and Cardiovascular Diseases, Chinese Academy of Medical Sciences, Beijing, China; ^3^Department of Pediatrics, People's Hospital of Wuhan University, Hubei, China; ^4^Department of Pediatrics, Children's Hospital of Kaifeng, Henan, China; ^5^Key Laboratory of Cardiovascular Sciences, Ministry of Education, Beijing, China

**Keywords:** baroreflex sensitivity, heart rate variability, orthostatic hypertension, orthostatic intolerance, pediatrics

## Abstract

There are no prior publications or submissions with any overlapping information, including studies and patients. The study data have not been presented as an abstract or poster before the submission.

**Objectives:** The study was conducted to analyze the changes of baroreflex sensitivity and heart rate variability from supine to upright standing in children and adolescents with orthostatic hypertension to explore whether and how the autonomic nerve regulation was involved in the development of pediatric orthostatic hypertension.

**Methods:** This case-control study included twenty-five children with orthostatic hypertension (the patient group) and twenty-six healthy controls (the control group). All subjects underwent a standing test, during which their hemodynamic parameters were continuously monitored by a Finapres Medical System, and baroreflex sensitivity and heart rate variability were calculated.

**Results:** The demographic characteristics, supine baroreflex sensitivity, and supine heart rate variability including time domain and frequency domain indices did not differ between the patients with orthostatic hypertension and healthy subjects (*P* > 0.05). However, a more obvious drop of baroreflex sensitivity and a greater increase of low frequency/high frequency ratio from supine to upright were observed in subjects with orthostatic hypertension compared with those in the healthy children (*P* < 0.001 and *P* < 0.01, respectively). Changes of baroreflex sensitivity were negatively related to mean arterial pressure changes from supine to upright in all subjects (*P* < 0.01), and the increases in low frequency/high frequency ratio from supine to standing were positively correlated with those in mean arterial pressure in the study subjects (*P* < 0.001).

**Conclusion:** Upright sympathetic overactivation is associated with pediatric orthostatic hypertension.

## Introduction

Orthostatic hypertension (OHT) refers to a significant blood pressure (BP) elevation in the upright position compared to supine or sitting position, which reflects abnormal regulation of BP during postural changes. Streeten et al. (1) first put forward the concept of OHT and studied its pathogenesis in the 1980s. Subsequently, multiple studies in adults with OHT show that OHT is seen in the elderly with essential hypertension or diabetes and patients with dysautonomias and also occurs in young adults with normal supine BP (2–5). Furthermore, OHT is closely related to the subsequent cardiovascular and cerebrovascular diseases and central nervous system damage, and it was regarded as a new risk factor for cardiovascular and cerebrovascular diseases in adults (3, 6–10). Therefore, increasing attention has been paid to OHT.

While, in adolescents and children, OHT has been recognized recently and it is now considered as an important cause of orthostatic intolerance (OI) (11–13). Our research group reported OHT in children for the first time in 2012. We discovered that most children with OHT were in the period of puberty, with OI symptoms as their main clinical manifestations, such as dizziness, headache or even syncope, etc (11). Kang et al. conducted a head-up tilt (HUT) test on 2,089 children with unexplained syncope, headache, dizziness, chest tightness, and sighing, and found that the prevalence of OHT was high in these children in the middle-south part of China (12). The abovementioned symptoms were often induced by postural change from supine to upright or prolonged standing (11, 13). The recurrent symptoms of OHT greatly impact on the academic performance and daily life in children and adolescents (11). Moreover, previous studies showed that OHT in young adults was associated with elevated risk of suffering from essential hypertension in the future (5, 14), which also drew focus on adolescents and children with OHT. However, up to now, the mechanism for pediatric OHT is poorly understood (13, 15, 16).

OHT stands for the hypertension occurring from supine to upright, and the autonomic nervous system regulates and maintains BP and heart rate (HR) during postural changes via baroreceptors (17). Hence, baroreflex sensitivity (BRS) and heart rate variability (HRV) are two widely accepted measures to assess the autonomic activity, and spectral analysis of HRV is used to reflect the balance between sympathetic and vagal tone (18). However, the changes in BRS and HRV indices from supine to upright in children and adolescents with OHT have not been yet clear.

Therefore, this study was aimed to examine the possible changes in BRS and HRV indices from supine to upright in children and adolescents with OHT, and reveal the role of autonomic regulation in the development of pediatric OHT.

## Methods

### Subjects

This case-control study enrolled 25 children with OHT (the OHT group) and 26 healthy children (the control group). The OHT group included 11 girls and 14 boys, from age 8 to 17 (12.5 ± 0.5) years old. All patients with OHT were admitted to the Department of Pediatrics at Peking University First Hospital from October 2015 to June 2019 with OI symptoms as their chief complaints and were diagnosed with OHT according to the published guidelines (19, 20). Specifically, the diagnostic criteria of OHT were as follows: mainly occurs in older children; associated with predisposing factors in most patients, such as prolonged standing, emotional stress, and crowded or stuffy environment; often associated with OI symptoms after upright; with a positive HUT test or standing test result (see *Standing test*); and exclusion of other diseases that cause OI symptoms (19, 20). The control group consisted of 12 girls and 14 boys, aged from 10 to 14 (12.0 ± 0.3) years old. They were recruited from elementary and junior high schools in two cities of China. They were considered healthy based on the medical history, physical examination and the standing test, and none of them had experienced OI symptoms within 3 months of the enrollment in the study.

### Standing Test

All subjects underwent a standing test. Before the test, all subjects were confirmed as not having any structural heart disease, arrhythmias, or neurologic disease and not taking any medication or food that might have influence on autonomic nervous function. The test was conducted in a quiet and dimly lit room. The children were asked to lay supine on the testing bed for 10 min, then stand upright on their own for another 10 min, and still then return to the supine position (19, 20) at the termination of the test. During the test, all subjects were asked to remain silent and breathe normally.

A positive OHT response to the standing test was defined as follows: normal supine BP; during the initial 3 min of the standing test, increased systolic BP (SBP)≥ 20 mmHg and/or increased diastolic BP (DBP) ≥ 25 mmHg (in children 6–12 years old) or ≥ 20 mmHg (in adolescents 13–18 years old) from supine to upright standing; or during upright standing, BP ≥ 130/90 mmHg (in children 6–12 years old) or ≥ 140/90 mmHg (in adolescents 13–18 years old) (19, 20).

### Data Recording and Analysis

During the standing test, HR, BP, and standard three-lead electrocardiograph were taken and recorded with a Dash 2000 Multi-lead Physiological Monitor (General Electric, New York, NY, USA). The last BP measured in supine position and the BP measured at 3 min after standing served as supine BP and upright BP, respectively. R-R interval (RRI) data obtained from electrocardiograph were visually reviewed, and five-min segments free of ectopic beats and artifacts severally in supine and upright position were used for further analysis.

Meanwhile, the Finapres Medical System (Finometer PRO, FMS Company, Netherlands) was applied to continuously and non-invasively record beat-to-beat BP data during the standing test and calculating hemodynamic parameters and BRS afterwards. Using a finger plethysmogram, beat-to-beat BP measures of SBP, DBP, and pulse intervals were collected. Then, stroke volume (SV) was computed with the model flow method. HR was calculated as the inverse of the pulse interval, and cardiac output (CO) was calculated as HR multiplying SV. Mean arterial pressure (MAP) was computed as the true integral of the arterial pressure wave over one beat, and total peripheral vascular resistance (TPVR) was calculated from MAP divided by CO. Average CO and TPVR taken from the last 1 min of the supine period was regarded as the supine values, and the upright CO and TPVR were computed as a 1-min average in the third minute after standing. The reliability of beat-to-beat BP measurements was validated using a Finapres system (21).

Cross-correlation was used to derive a form of sequential BRS. The correlation between interbeat interval and beat-by-beat SBP, with delays of 0–5 s for interval, was sampled and computed. When the correlation was significant at *P* < 0.01, the slope between SBP and interbeat interval was documented as one BRS value (22). Average supine and upright BRS values were taken consistent with the rule of CO and TPVR described above.

Five-min segments of RRI data were used to calculate time domain and frequency domain parameters of HRV. Standard deviation of RRI (SDNN) and root mean square of successive differences (RMSSD) of RRI were analyzed. Original RRI series was altered to equidistantly sampled sequence by a cubic spline interpolation method. Fast Fourier transformation was then used to obtain power spectral density (PSD) functions with Welch's periodogram method. Total power (TP) in the frequency range from 0.0 to 0.4 Hz was composed of very low frequency (VLF, 0.0–0.04 Hz), low frequency (LF, 0.04–0.15 Hz) and high frequency (HF, 0.15–0.4 Hz) bands. Powers of each band were computed as integrals under the respective PSD functions. The ratio between normalized LF power and HF power was calculated as LF/HF ratio.

### Statistical Analysis

Statistical analyses were carried out with SPSS 20.0 software (SPSS, Chicago, IL, USA). All data are expressed as means ± SE, and *P* < 0.05 (2-tailed) indicated a statistically significant difference. The independent *t* test was applied for the comparisons between groups in the same position, and the paired *t* test was utilized to compare the corresponding parameters before and at standing for the same person. The Chi-squared test was used to compare categorical variables. Covariance analysis was adopted to compare all autonomic measures (BRS and HRV) after adjusting for gender, age and body mass index (BMI). Pearson correlation analysis was used for linear correlations and partial correlation analysis was adopted to correct the influence of gender, age, and BMI.

## Results

### Subject Characteristics

The sex ratio, age, height, weight, or BMI between the subjects in OHT and in control groups did not significantly differ (*P* > 0.05, Table 1).

**Table 1:** Demographic characteristics of the OHT group and control group.

**Groups**	***n***	**Gender (M/F)**	**Age (years)**	**Height (cm)**	**Weight (kg)**	**BMI (kg/m^**2**^)**
OHT	25	14/11	12.5 ± 0.5	159.0 ± 3.2	53.5 ± 3.0	20.7 ± 0.6
Control	26	14/12	12.0 ± 0.3	158.4 ± 1.6	48.1 ± 1.5	19.1 ± 0.5
t /χ^2^	–	0.024	0.810	0.166	1.599	1.925
*P*-value	–	0.877	0.424	0.869	0.119	0.060

*Values are expressed as means ± SE. BMI, body mass index; OHT, orthostatic hypertension*.

### Changes in Hemodynamic Parameters During the Standing Test

**Figure 1:**
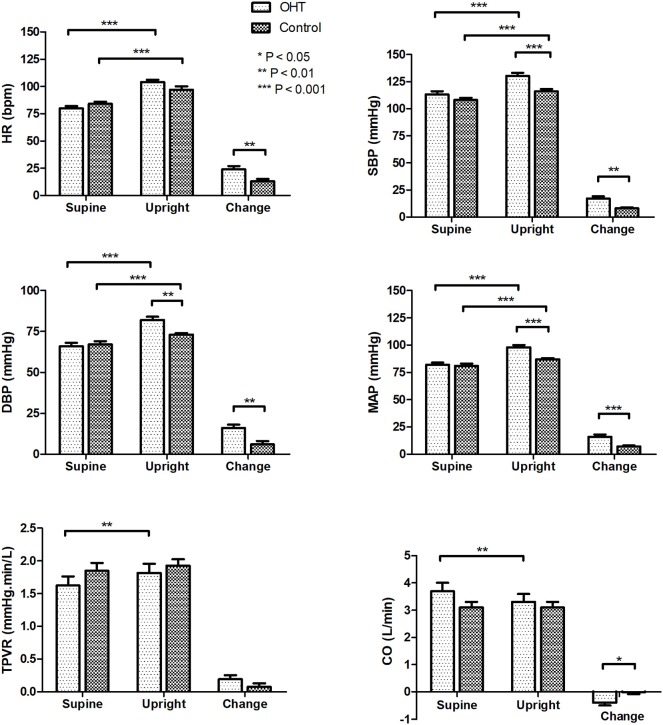
Hemodynamic changes of the study subjects during the standing test. Values are means ± SE. *P < 0.05, **P < 0.01, ***P < 0.001. OHT, orthostatic hypertension; HR, heart rate; SBP systolic blood pressure; DBP, diastolic blood pressure; MAP, mean arterial pressure; TPVR, total peripheral vascular resistance; CO, cardiac output.

As seen in Figure 1, in the supine position, all of the hemodynamic measures did not differ between the patients with OHT and the control group. While, HR, SBP, DBP, and MAP increased after standing in both groups (*P* < 0.001 for all variables). However, at 3 min of standing, the patients with OHT exhibited significantly higher SBP, DBP, and MAP than the control subjects (*P* < 0.01 for DBP, and *P* < 0.001 for SBP, and MAP). In addition, from supine to standing, OHT patients experienced an obvious decrease in CO (*P* < 0.01) and a marked increase in TPVR (*P* < 0.01), while the CO and TPVR of the control group did not show any significant changes upon standing (*P* > 0.05).

### Changes in BRS and HRV Measures During the Standing Test

**Figure 2:**
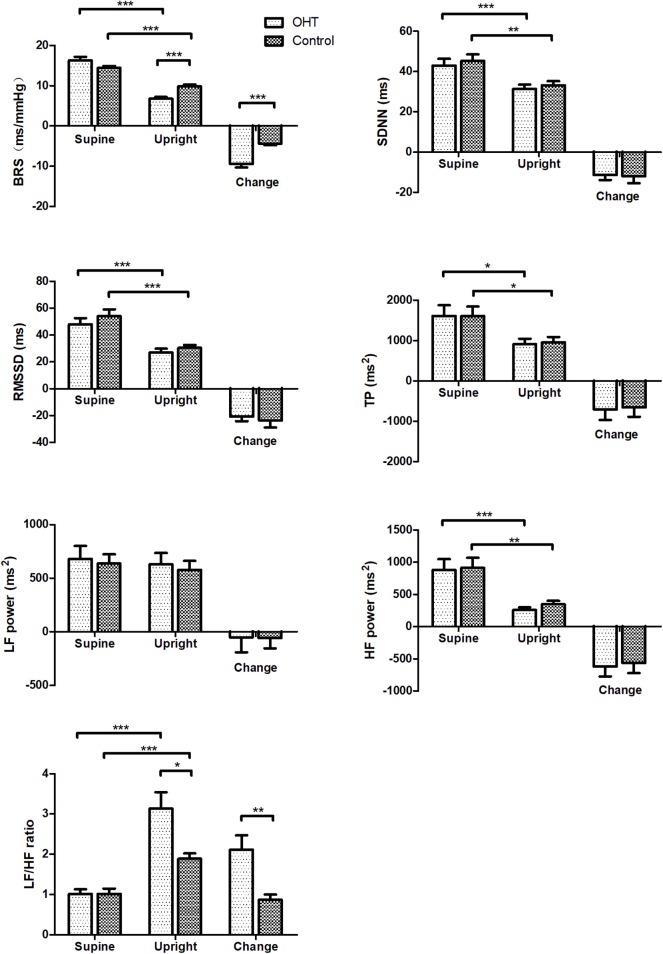
Changes in BRS and HRV measures of the study subjects during the standing test. Values are means ± SE. *P < 0.05, **P < 0.01, ***P < 0.001. OHT, orthostatic hypertension; BRS, baroreflex sensitivity; SDNN, standard deviation of R-R intervals; RMSSD, root mean square of successive differences; TP, total power; LF, low frequency; HF, high frequency.

BRS and HRV changes from supine to upright are shown in Figure 2. At rest, there were no statistical differences in BRS and HRV estimates between the two groups. After standing, the BRS of the OHT patients decreased more significantly than that of the controls (*P* < 0.001), and the upright BRS in the OHT group was significantly lower than that in the control group after controlling for gender, age and BMI (*P* < 0.001). As for HRV indices, SDNN, RMSSD, TP, and HF power markedly decreased upon standing in all subjects, but at standing, SDNN, RMSSD, TP, LF power, or HF power did not significantly differ between the two groups. While, the elevation in LF/HF ratio from supine to upright was significantly greater in the OHT subjects compared with the healthy children, after controlling for gender, age and BMI (*P* < 0.01). Specific values in Figures 1, 2 are displayed in the Supplementary Table 1.

### Association of Changes in BRS and LF/HF Ratio With the Changes in Blood Pressure From Supine to Upright

**Figure 3:**
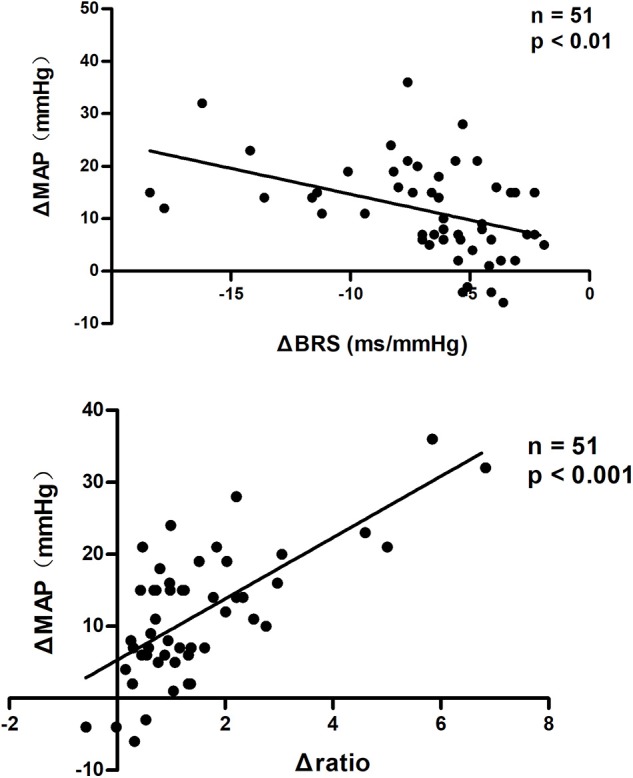
Pearson correlation analysis of changes in BRS and LF/HF ratio with BP changes from supine to upright. MAP changes from supine to upright were negatively correlated with BRS changes and positively correlated with LF/HF ratio changes in all subjects. BRS, baroreflex sensitivity; MAP, mean arterial pressure. ΔBRS, upright BRS – supine BRS; ΔMAP, upright MAP – supine MAP; Δratio, upright LF/HF ratio – supine LF/HF ratio.

Pearson correlation analysis showed that changes in BRS were negatively correlated with changes in MAP from supine to upright in all subjects (*P* < 0.01, Figure 3). While, changes in LF/HF ratio were positively correlated with the MAP elevations from supine to standing (*P* < 0.001, Figure 3). In partial correlation analysis, the correlation coefficients were −0.442 and 0.709, respectively, after adjusting for gender, age and BMI.

## Discussion

For the first time in this study, we explored the alterations in the autonomic nervous tone from supine to standing through BRS and HRV indices in children and adolescents with OHT and healthy controls, and found a significant drop in BRS but an obvious rise in LF/HF ratio from supine to upright in the patients with OHT after controlling for confounding variables. The results suggested a sympathetic overdrive upon standing in pediatric OHT patients and greatly contributed to the understanding of the mechanisms for pediatric OHT.

The mechanisms responsible for the development of pediatric OHT have not been clear. Kario et al. (8) measured changes in plasma norepinephrine (NE) levels in OHT patients before and after tilting. In their study, after tilting, the plasma NE level was significantly higher in the OHT group than that in the controls, while the supine plasma NE levels were comparable between the two groups. Moreover, α-receptor blockers have been shown to be capable of reducing the upright BP without effecting baseline BP in clinical studies (8, 23). Based on the above facts, we hypothesized that the abnormal autonomic nervous system control is likely associated with OHT. Therefore, the present study was designed to analyze the possible involvement of autonomic nervous dysfunction in the development of pediatric OHT by detecting the changes in autonomic measures from supine to upright. BRS and HRV are two commonly used measures of autonomic nerve function. Baroreflex is essential in the instant regulation of BP (24, 25), and LF/HF ratio reflects the predominant component among sympathetic and vagal tone (18). Normally, BRS and RRI variability decrease but LF/HF ratio increases after standing (17, 18, 26). However, both the decrease in BRS and the increase in LF/HF ratio from supine to upright were significantly greater in pediatric patients with OHT than those in controls. The reduction of BRS is associated with the sympathetic activation (17, 26, 27), and the increased LF/HF ratio indicates an obvious sympathetic predominance. Therefore, the significant drop of BRS and the remarkably increased LF/HF ratio at standing in the OHT patients demonstrated sympathetic overactivity in adolescents with OHT when upright. Furthermore, we found that both the changes in BRS and the changes in LF/HF ratio from supine to upright were linearly correlated with the changes in MAP in all subjects, which indicated that the severity of BP elevation was related to the degree of sympathetic activation. In addition, it is worth noting that we recorded a decrease in CO and an increase in TPVR after standing in adolescents with OHT, while CO and TPVR did not change significantly in healthy controls from supine to upright. The decrease in CO upon upright might be the trigger for sympathetic overactivation in OHT patients. The previous studies showed that, after wearing inflatable pressure suits to increase returned blood volume, the upright DBP of the OHT patients was lower than before (1). Besides, we controlled the gender, age and BMI when analyzing autonomic measures to exclude their influence on autonomic activity (23, 24, 28).

In this research, no significant differences in supine BRS and RRI variability were found between the individuals with and without OHT. In contrast to our results, Yoshinari et al. (29) reported that the coefficient of variation of the RRI at rest was higher in diabetic patients with OHT than in diabetic patients without OHT, suggesting an increased baseline BRS level in OHT patients. However, they only recorded the RRI for 200 beats on the electrocardiogram in the supine position, and did not compute BRS directly. More importantly, the participants were quite different from ours, since diabetes mellitus affected autonomic nervous function itself. In a pilot study on children with OI, Wagoner et al. (30) found that there was no significant difference in BRS measures between OI and non-OI subjects in the supine position, and BRS decreased in OI subjects upon standing, but they did not subgroup the OI subjects. As for HRV measures, Yang et al. analyzed Holter ECG results of children with OHT, and found that LF/HF ratio in the OHT group was higher than that in the control group (31). However, they did not show the comparisons of HRV measures among different positions, including the upright position.

To date, there have been no reports of medication therapy for pediatric OHT since the mechanism for pediatric OHT is poorly understood (13). However, despite suffering from recurrent OI symptoms, children with OHT might have increased potential of developing hypertension in the future as the frequent fluctuations of BP might damage the function of the vascular wall and endothelial cells (32). Therefore, children and adolescents with OHT urgently need assessment for the necessity of medication. Our results provided a potential therapeutic target, an excessive activation of the sympathetic nervous system, though more research evidence is needed in the future.

The limitations of our study would be that first, the non-invasive methods (BRS and HRV analysis) that we used for assessing the autonomic nervous function, due to the difficulties in the implementation of invasive methods in children, are not the most direct. Muscle sympathetic nerve activity (MSNA) detected by microneurography is another reliable way of evaluating sympathetic activity, which is more direct, but it is hard to conduct and not included in the present study due to the invasiveness of testing (33). The coherence between HRV and MSNA spontaneous variability at rest and during orthostatic challenge has been proved before, and HRV is easier to obtain compared with MSNA (34). Second, the sample size in our study is relatively small, which might lead to the increase of sampling error. Therefore, multiple-center based and large-sample sized studies will be needed in the future to further validate the role of autonomic nervous regulation in the development of pediatric OHT.

In conclusion, our study provided new insight into the vital role of sympathetic hyper-activation upon standing in pediatric OHT. The data would greatly further the understanding of the mechanisms for OHT in children and adolescents.

## Data Availability Statement

The datasets generated for this study are available on request to the corresponding author.

## Ethics Statement

The studies involving human participants were reviewed and approved by The Ethics Committee of Peking University First Hospital. Written informed consent to participate in this study was provided by the participants' legal guardian/next of kin.

## Author Contributions

JD, BH, and CTan contributed to the conception and design of the study, revise of the manuscript, and final approval of the version to be published. YH, YuW, YaW, YJ, ZH, CTao, CTan, and HL analyzed and interpretated the data, and drafted the article. YyW, BH, YaW, ZH, CTao, and HL undertook the test and acquired the data. JD, YJ, and CTan revised the manuscript critically for important intellectual content.

### Conflict of Interest

The authors declare that the research was conducted in the absence of any commercial or financial relationships that could be construed as a potential conflict of interest.
