# Supplementary material for: Sympathetic Overactivation From Supine to Upright Is Associated With Orthostatic Hypertension in Children and Adolescents
Source: Front Pediatr. 2020 Feb 21;8:54. doi: 10.3389/fped.2020.00054 (PMC7047410; doi:10.3389/fped.2020.00054)
Supplement: Supplementary file 1 [file Table_1.docx]

**Table 1. Hemodynamic and autonomic measures of the study subjects during the standing test.**

|  | OHT | | | Control | | |
| --- | --- | --- | --- | --- | --- | --- |
|  | **Supine** | **Upright** | **Change** | **Supine** | **Upright** | **Change** |
| HR (bpm) | 80 ± 2 | 104 ± 2 | 24 ± 3 | 84 ± 2 | 97 ± 3 | 13 ± 2 |
| SBP (mmHg) | 113 ± 3 | 130 ± 3 | 17 ± 2 | 108 ± 2 | 116 ± 2 | 8 ± 1 |
| DBP (mmHg) | 66 ± 2 | 82 ± 2 | 16 ± 2 | 67 ± 2 | 73 ± 1 | 6 ± 2 |
| MAP (mmHg) | 82 ± 2 | 98 ± 2 | 16 ± 2 | 81 ± 2 | 87 ± 1 | 7 ± 1 |
| CO (L/min) | 3.7 ± 0.3 | 3.3 ± 0.3 | -0.4 ± 0.1 | 3.1 ± 0.2 | 3.1 ± 0.2 | -0.01 ± 0.07 |
| TPR (mmHg.min/L) | 1.623 ± 0.138 | 1.816 ± 0.142 | 0.193 ± 0.063 | 1.849 ± 0.120 | 1.928 ± 0.097 | 0.079 ± 0.054 |
| BRS (ms/mmHg) | 16.3 ± 0.9 | 6.8 ± 0.4 | -9.5 ± 0.8 | 14.4 ± 0.5 | 9.9 ± 0.4 | -4.4 ± 0.3 |
| SDNN (ms) | 43.0 ± 3.3 | 31.5 ± 2.1 | -11.4 ± 2.5 | 45.3 ± 3.3 | 33.2 ± 2.1 | -12.1 ± 3.3 |
| RMSSD (ms) | 47.8 ± 4.7 | 27.1 ± 2.7 | -20.8 ± 3.5 | 54.0 ± 5.2 | 30.4 ± 2.2 | -23.6 ± 5.3 |
| TP (ms^2^) | 1616 ± 265 | 912 ± 137 | -704 ± 264 | 1614 ± 231 | 961 ± 130 | -653 ± 238 |
| LF power (ms^2^) | 681 ± 119 | 628 ± 106 | -52 ± 137 | 637 ± 85 | 578 ± 82 | -58 ± 96 |
| HF power (ms^2^) | 876 ± 170 | 255 ± 39 | -621 ± 154 | 909 ± 159 | 345 ± 54 | -565 ± 158 |
| LF/HF ratio | 1.01 ± 0.12 | 3.13 ± 0.41 | 2.12 ± 0.35 | 1.02 ± 0.13 | 1.89 ± 0.13 | 0.87 ± 0.13 |

Note: Values are expressed as means **±** SE. OHT, orthostatic hypertension; HR, heart rate; SBP systolic blood pressure; DBP, diastolic blood pressure; MAP, mean arterial pressure; CO, cardiac output; TPR, total peripheral resistance; BRS, baroreflex sensitivity; SDNN, standard deviation of R-R intervals; RMSSD, root mean square of successive differences; TP, total power; LF, low frequency; HF, high frequency.
